# Clinical and genetic diagnosis of Cowden syndrome: A case report of a rare *PTEN* germline variant and diverse clinical presentation

**DOI:** 10.1097/MD.0000000000032572

**Published:** 2023-01-06

**Authors:** Hironori Arai, Kiwamu Akagi, Ayako Nakagawa, Yasuhide Onai, Yoshikazu Utsu, Shinichi Masuda, Nobuyuki Aotsuka

**Affiliations:** a Department of Hematology and Oncology, Japanese Red Cross Narita Hospital, Narita, Japan; b Department of Molecular Diagnosis and Cancer Prevention, Saitama Cancer Center, Saitama, Japan; c Department of Breast Surgery, Japanese Red Cross Narita Hospital, Narita, Japan.

**Keywords:** Cowden syndrome, *PTEN*, Lhermitte–Duclos disease, dural arteriovenous fistula, surveillance, germline, variant

## Abstract

Cowden syndrome is a rare autosomal dominant disease characterized by the development of hamartomas and increased risks of other tumors, including breast, thyroid, and uterine cancers. Most patients with Cowden syndrome show mutations of the phosphatase and tensin homolog (PTEN) gene on chromosome 10; however, some patients with mutations do not show clinical symptoms, while patients with clinical symptoms may not have detectable PTEN mutations.

**Case presentation:**

A 39-year-old woman with macrocephaly had previously been diagnosed with Cowden syndrome at another hospital, when she presented with the onset of breast cancer. A wide variety of complications were detected, including cerebellar tumors treated by resection, hydrocephalus, and multiple polyps in the stomach and large intestine. She was further diagnosed with adult-onset Lhermitte–Duclos disease as a complication of Cowden syndrome. She subsequently developed a dural arteriovenous fistula treated by transvenous embolization. After transfer to our hospital, she developed adenomatous goiter treated by resection, recurrent breast cancer treated with hormonal therapy, and multifocal oral mucosal papillomatosis. Her older sister had previously been diagnosed with Cowden syndrome and her father was undiagnosed but had macrocephaly, hydrocephalus, and multifocal oral mucosal papillomatosis, suggestive of Cowden syndrome. After consultation with a genetic specialist, analysis of the PTEN gene showed a rare but likely pathogenic germline c.801 + 2T>A variant located at the splice donor site of intron 7. The patient’s clinical diagnosis of Cowden syndrome was accordingly confirmed by the genetic findings. Appropriate surveillance procedures were put in place to detect any further tumors.

**Conclusions:**

The clinical symptoms of Cowden syndrome do not always correlate with the genetic results. However, recent improvements in genetic testing suggest the importance of diagnosing this disease using both clinical and genetic approaches, in collaboration with genetic experts, to ensure an accurate diagnosis and appropriate surveillance for malignant tumors.

## 1. Introduction

Cowden syndrome is a rare autosomal dominantly inherited disease associated with the phosphatase and tensin homolog (*PTEN*) gene. It is characterized by hamartomatous lesions in the gastrointestinal tract, skin, mucus membranes, breast, thyroid gland, endometrium, and brain. Macrocephaly and multiple cutaneous and mucosal lesions are frequently present. However, some cases without a *PTEN* variant are diagnosed with clinical Cowden syndrome, while other cases with a *PTEN* variant do not meet the clinical diagnostic criteria. We report on a case of Cowden syndrome that was confirmed genetically by the presence of a *PTEN* variant in the splice site and clinically by several typical complications.

## 2. Case presentation

### 2.1. Clinical course

**Figure 1.**
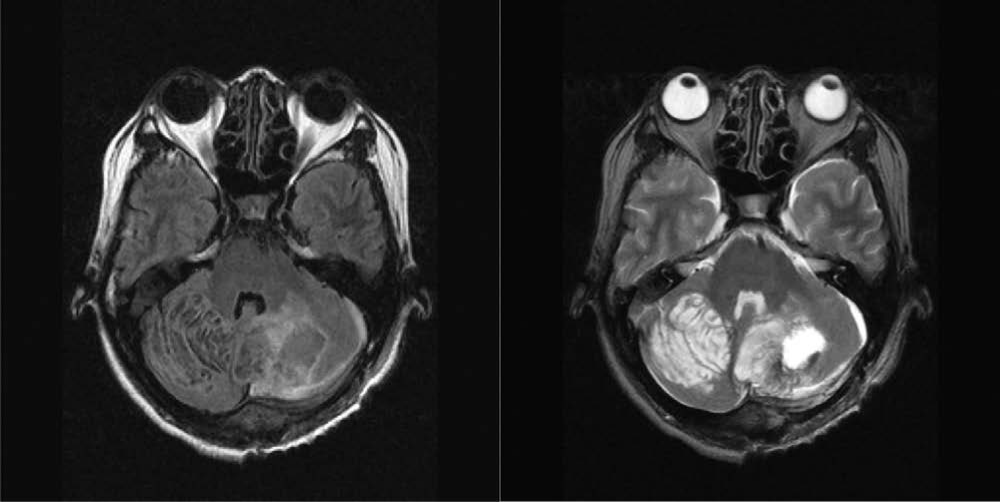
Magnetic resonance images of bilateral cerebellar hamartomas in the patient aged 40 years, after left tumor resection. Left: T1-weighted image, right: T2-weighted image.

The patient was a 50-year-old woman. She had been recognized as having a slightly large head since childhood, but had never been diagnosed with mental retardation. At the age of 39, her doctor detected bilateral breast masses and performed a biopsy, which led to a diagnosis of bilateral invasive ductal carcinoma of the breast. A whole-body examination revealed cerebellar tumors and hydrocephalus, and she was diagnosed with Lhermitte–Duclos disease as a complication of Cowden syndrome (Fig. 1). Multiple polyps were found in the stomach and large intestine. She underwent left cerebellar tumor resection at the age of 40 years and the pathological results indicated hamartomas.

She had resigned from her job to focus on her treatment, but subsequently experienced depression because she could not find another job after her surgery. At the age of 41 years, she was referred to a psychiatry department where she was diagnosed with depression and started on medication, with gradual relief of her depressed mood.

**Figure 2.**
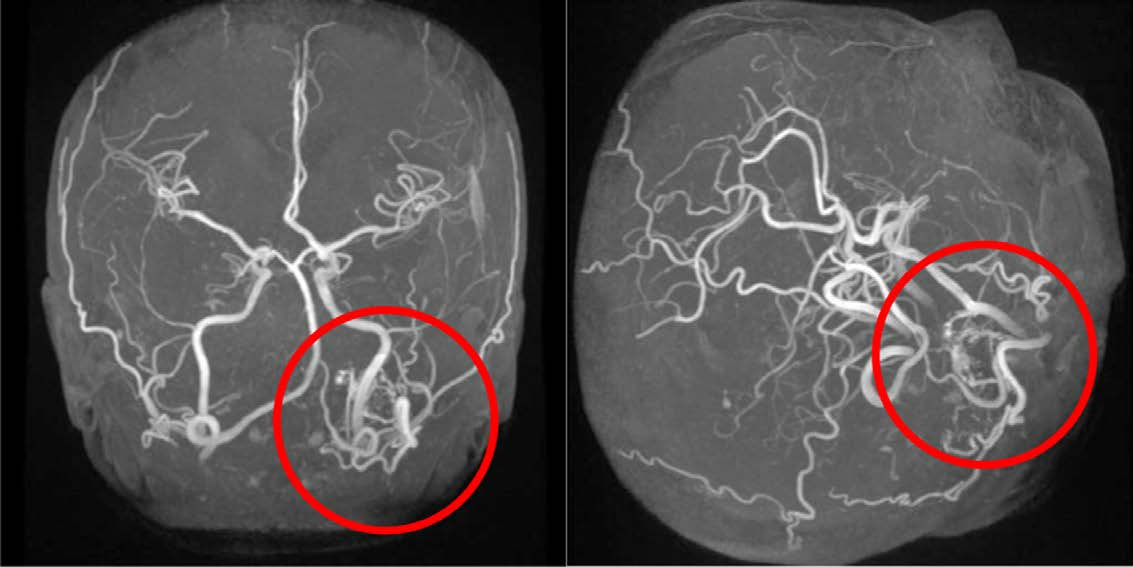
Frontal and lateral images of cerebral angiogram. Dural arteriovenous fistula in the patient aged 43 years, before transvenous embolization.

At the age of 43, she developed right upper and lower extremity incomplete hemiplegia and close examination revealed a dural arteriovenous fistula (Fig. 2). She subsequently underwent transvenous embolization on 3 occasions.

At the age of 45, she changed her residence and was referred to the Departments of Breast Surgery, Neurosurgery, and Psychiatry at the Japanese Red Cross Narita Hospital for follow-up. She underwent right lobe resection by otolaryngologists in our hospital at age 46, because of a mass in the thyroid gland. The pathological result was an adenomatous goiter. In October of the same year, she was diagnosed with recurrent breast cancer with left-rib metastasis, and started hormonal therapy. She was referred to our Department of Oncology for continuous surveillance at age 50 years.

### 2.2. Family history

**Figure 3.**
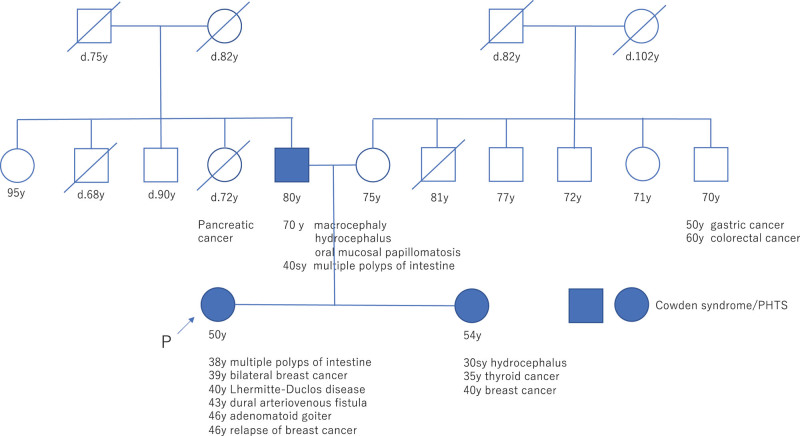
Family tree. The patient’s sister had previously been diagnosed with Cowden syndrome. Her father was strongly suspected to have Cowden syndrome based on his medical history.

Regarding the patient’s family history (Fig. 3), her older sister had been clinically diagnosed with Cowden syndrome at another hospital. Her father was undiagnosed, but the obtained information indicated that he had macrocephaly, hydrocephalus, and multifocal oral mucosal papillomatosis, and was therefore considered likely to have Cowden syndrome, as defined by the National Comprehensive Cancer Network (NCCN) diagnostic criteria.

### 2.3. Physical and laboratory findings

The patient’s physical and laboratory findings at presentation showed clear consciousness. The right lobe of her thyroid and bilateral breasts had been resected. Her head circumference was 61 cm. She had multifocal oral mucosal papillomatosis. Her blood and urine findings and abdominal ultrasound results were normal.

### 2.4. Progress after consultation

**Figure 4.**
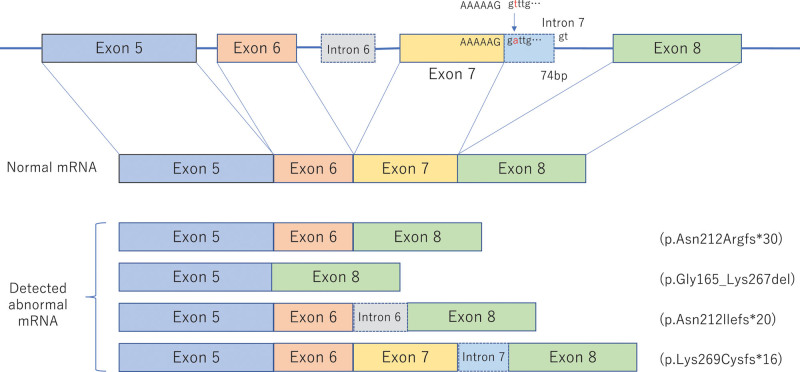
Splicing abnormality of *PTEN* gene detected in the reported case. *PTEN* = phosphatase and tensin homolog.

Neither the patient nor her sister had any children and there was no possibility of further inheritance because both had reached menopause. However, it was necessary to gain a better understanding of the patient’s condition from a genetic specialist and to consider the future course of action. We considered transferring the patient to another hospital that had a genetic specialist, but she preferred to be monitored at our hospital. The attending doctor and the patient therefore consulted a genetic specialist at another hospital and surveillance was carried out at our hospital under his guidance. Analysis of the *PTEN* gene showed a likely pathogenic c.801 + 2T>A variant, leading to a genetic diagnosis of Cowden syndrome (Fig. 4). Surveillance for uterine cancer was requested at the Department of Obstetrics and Gynecology, and periodic lower gastrointestinal endoscopy, urinalysis, and abdominal ultrasound were planned to detect renal or colorectal cancer.

The c.801 + 2T>A variant is considered as “likely pathogenic” according to the American College of Medical Genetics, because it is located at the splice donor site of intron 7 (PVS1). It a very rare variant that is not registered in The Genome Aggregation Database in the general population, including in Japanese (PM2), but is registered as “pathogenic” in ClinVar. Primers were designed for exons 5 to 8 to cover exon 7, and splicing defects were detected by reverse transcription-polymerase chain reaction and ribonucleic acid (RNA) sequencing. Messenger RNA (mRNAs) lacking exon 7 were detected. mRNAs having a cryptic exon as part of the intron 6 sequence and mRNAs produced by splicing with a new donor site at “GT” 74 nucleotides downstream from the mutation site were also detected. Both of these splicing aberrations cause a frameshift that stops PTEN protein synthesis. These mRNAs were degraded by nonsense-mediated mRNA decay. In addition, deletion of exons 6 and 7 results in the synthesis of proteins with deletions from glycine 165 to lysine 267. These results suggested that alleles with these variants were unlikely to produce normal PTENs. Based on the above, the detected variant was considered to cause Cowden syndrome.

## 3. Discussion and conclusions

Cowden disease causes neoplasms in various organs of mesodermal and ectodermal origin throughout the human body, with a high rate of polyposis in the entire gastrointestinal tract, from the esophagus to the rectum. Cowden disease is thought to be caused by a germline variant of the *PTEN* gene on chromosome 10 (10q23.31) and is inherited in an autosomal manner. The frequency is estimated to be about 1 in 200,000, and its penetrance is high, with about 90% of cases showing some disease-specific signs by their late 20s.^[1,2]^ Its actual prevalence is thought to be higher, with many cases remaining undiagnosed^[3,4]^; however, its diagnosis rate has increased in line with the widespread use of genetic testing for *PTEN*. Nevertheless, recent studies showed that only approximately 80% of patients with clinically diagnosed Cowden syndrome had pathological variants, while 10% of patients had deletions, duplications, or nucleotide sequence abnormalities in promoter regions that were not detectable by conventional testing.^[3,5]^ In addition, some cases with symptoms similar to Cowden disease lack a germline variant of the *PTEN* gene. These cases have been reported to show germline variants of succinate dehydrogenase subunit X, p53-regulated deoxyribonucleic acid replication inhibitor, protein kinase B (*AKT*), phosphoinositide 3-kinase (PI3K, *PIK3CA*), and SEC23 homolog B, coat complex component (*SEC23B*), which are involved in the PI3K/AKT/mammalian target of rapamycin (mTOR) pathway, which in turn involves PTEN; however, the functions of other genes not related to this pathway are still unknown. Therefore, Cowden syndrome cannot be ruled out even in the absence of *PTEN* abnormalities, and the syndrome is currently diagnosed when the clinical criteria are met, even without identification of a pathological variant of *PTEN*. Other groups of diseases are defined as PTEN hypomethyloma syndrome (PHTS), such as Bannayan–Riley–Rubarkava syndrome and Proteus syndrome, and the presence of a pathological *PTEN* germline variant may not always lead to a correct diagnosis.

An accurate diagnosis of Cowden syndrome thus requires combined evaluation of the clinical and genetic findings. The current patient fulfilled the 4 major criteria for the diagnosis of Cowden syndrome according to the NCCN: breast cancer, adult-onset Lhermitte–Duclos disease, macrocephaly, multiple cutaneous and mucosal neuromas, and papillomatous lesions of the oral mucosa. They also met 3 minor criteria: esophageal glycogen acanthosis (Fig. 2), abnormal vasculature of the dural arteriovenous fistula, and adenomatous goiter. The presence of a pathologically significant germline mutation in the *PTEN* gene allowed both a clinical and genetic diagnosis of Cowden syndrome. Her father also had macrocephaly, hydrocephalus, and papillomatosis of the oral mucosa, suggesting that the patient may have inherited the disease from him, but no more upstream family history was available. It is possible that the father had a mutation in his *PTEN* gene and passed this on to his daughters.

The recent spread of novel genetic tests, such as comprehensive genomic profiling, means that germline *PTEN* gene variants have been found incidentally, leading to its detection in an increasing number of cases. The phenotype of Cowden disease varies with age: most adults present with Cowden syndrome, whereas children often present with macrocephaly, autism spectrum disorder, and Bannayan–Riley–Rubarkava syndrome. The term Cowden syndrome/PHTS is often used, given that most patients with PHTS present with Cowden syndrome.

Hamartomas and carcinomas are thought to be caused by the dysfunctional mutated PTEN product, which leads to activation of the PI3K/AKT/mTOR pathway, as well as by other genetic abnormalities.^[6]^ However, the detailed mechanism by which dysfunctional PTEN contributes to tumorigenesis is still unclear but may involve inactivation of both alleles of the *PTEN* gene by 2-hit, haploinsufficiency, a dominant-negative effect, deoxyribonucleic acid methylation of promoter regions, and abnormal expression of micro RNAs and long non-coding RNAs that regulate *PTEN*.^[7–10]^ Pathogenic germline variants, including missense and nonsense variants, splice sites, and deletions and insertions, have been reported in all 9 exons in the *PTEN* gene,^[11–13]^ and are often overexpressed, especially in exons 5, 7, and 8.^[14–16]^ Specific pathological variants also occur in the intron region, causing exon skipping and alternative splicing, and the use of cryptic splice sites.^[17]^ We consider that the current variant produced various aberrant mRNAs, thus arresting PTEN protein synthesis; however, this variant has not yet been analyzed.

Patients with Cowden syndrome/PHTS are at high risk of malignant complications, including breast, thyroid, endometrial, and colorectal tumors, and renal cell carcinoma. It is therefore important to conduct appropriate surveillance to detect such diseases at an early stage, according to the NCCN guidelines.^[18]^ Surveillance for breast cancer includes breast self-examination after age 18 years, annual breast magnetic resonance imaging, and mammography and breast ultrasound every 6 months, starting after age 30 years or 5 to 10 years before than the lowest age of cancer incidence in the family. Patients should also receive annual cervical ultrasound examinations to detect thyroid cancer after age 16 years, annual internal examinations and transvaginal ultrasound to detect uterine cancer after age 30 years, lower gastrointestinal endoscopy to detect colorectal cancer after age 35 years or 5 to 10 years below the lowest age of cancer incidence in the family, and annual abdominal ultrasonography to detect renal cell carcinoma after age 40 years.

The current patient had already been treated for breast cancer, Lhermitte–Duclos disease, and adenomatous goiter prior to referral to our Department of Oncology, each of which had been passed on to our specialists from the previous doctors. However, there had been no interventions for renal cell carcinoma and endometrial carcinoma.

We considered that it was necessary for both the attending doctor and the patient to consult a genetic specialist and obtain advice, to understand the disease and plan future surveillance. The patient was receiving fulvestrant plus palbociclib as standard second-line therapy for recurrent breast cancer at the time of this report.

The characteristics of Cowden syndrome/PHTS-related cancers are not completely understood, but their common feature is an abnormality in the *PTEN* gene, and the growth rate, metastatic and invasive potential, and drug sensitivity of these cancers are thus expected to be similar to those in patients with somatic *PTEN* variants. In addition, drugs that inhibit the PI3K/AKT/mTOR pathway, which is downstream of the functioning PTEN protein, may be effective and are currently undergoing clinical trials.^[19]^

We report on a case of Cowden syndrome with a previously unreported pathological germline variant of the *PTEN* splice site. Although advances in genetic testing have improved the diagnosis rate of Cowden syndrome, it is important to diagnose the disease both clinically and genetically, because the symptoms are not always correlated with the genetic alteration. There is currently no effective treatment for Cowden syndrome, and the resulting tumors are still treated in the same way as sporadic tumors. However, appropriate surveillance in cooperation with genetic specialists enables the implementation of preventive measures and the early detection of malignant tumors. The treatment of hereditary tumors is expected to progress further in the future, and oncologists will accordingly need to improve their ability to treat hereditary tumors.

## Acknowledgments

We thank Susan Furness, PhD, from Edanz (https://jp.edanz.com/ac) for editing a draft of this manuscript.

## Author contributions

KA contributed to the conception and design of the work and substantively revised the manuscript. YO and AN treated the patient and provided treatment advice. YU, SM, and NA supervised the work.

**Conceptualization:** Kiwamu Akagi.

**Supervision:** Ayako Nakagawa, Yasuhide Onai.

**Writing – original draft:** Hironori Arai.

**Writing – review & editing:** Yoshikazu Utsu, Shinichi Masuda, Nobuyuki Aotsuka.
